# Editorial: Exoskeleton gait training

**DOI:** 10.3389/fnins.2025.1705522

**Published:** 2025-10-20

**Authors:** Yury Ivanenko, Mikhail A. Lebedev

**Affiliations:** ^1^Laboratory of Neuromotor Physiology, IRCCS Fondazione Santa Lucia, Rome, Italy; ^2^Ural Federal University, Yekaterinburg, Sverdlovsk Oblast, Russia; ^3^Faculty of Mechanics and Mathematics, Lomonosov Moscow State University, Moscow, Russia; ^4^Sechenov Institute of Evolutionary Physiology and Biochemistry of the Russian Academy of Sciences, St Petersburg, Russia

**Keywords:** locomotion, exoskeletons, neuromodulation, adaptive control, neurorehabilitation

## Introduction

**Figure 1:**
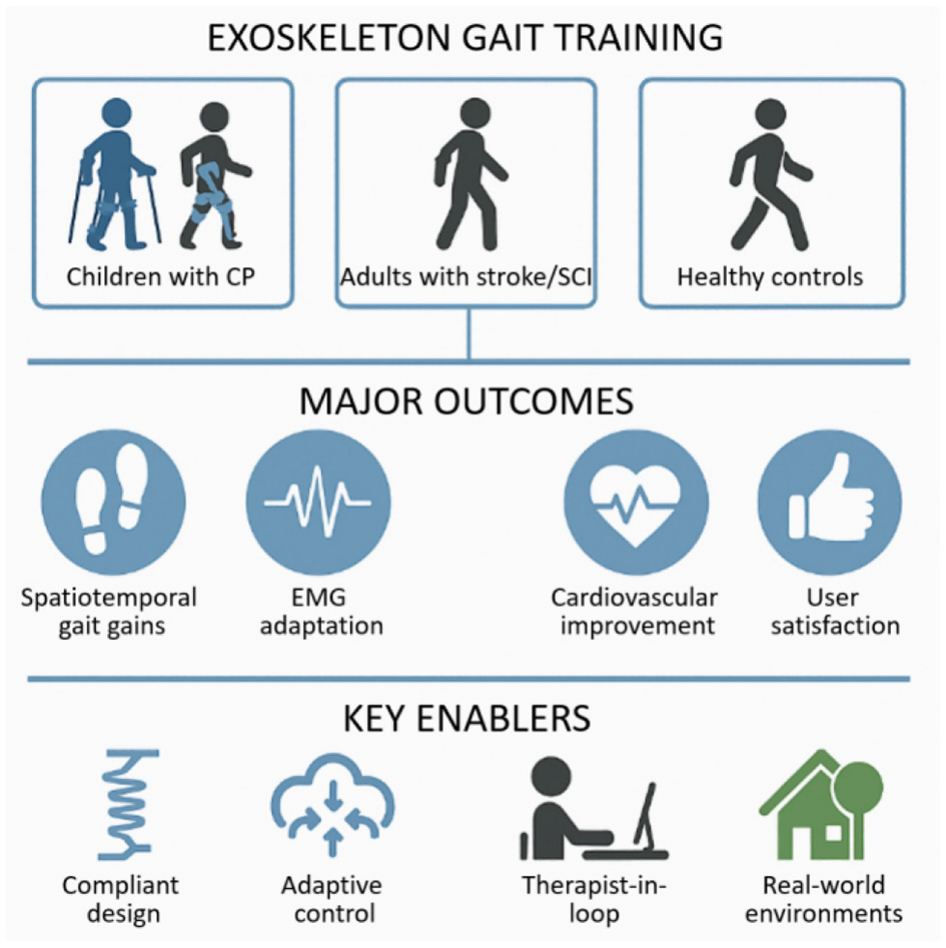
Overview of key themes emerging from the Research Topic “*Exoskeleton Gait Training*.” Populations studied across the included papers comprised children with cerebral palsy (CP), adults with stroke and spinal cord injury (SCI), and healthy participants. Primary outcomes included improvements in spatiotemporal gait parameters, neuromuscular (EMG) adaptation, cardiovascular benefits, and increased user satisfaction. Enabling factors that contributed to these outcomes were compliant device design, adaptive control strategies, therapist-guided sessions, and training conducted in real-world environments.

Powered lower-limb exoskeletons increasingly show promise for restoring mobility, inducing neuromuscular plasticity, and enriching quality of life in neurological populations [e.g., stroke, spinal cord injury (SCI), cerebral palsy (CP)]. However, key challenges persist, namely: understanding mediators of training effectiveness, refining control paradigms for personalization, and assessing real-world deployment. With contributions spanning clinical trials, biomechanics, control mechanisms, and feasibility studies (Figure 1), this Research Topic illuminates progress and remaining hurdles.

## Mechanisms and control: insights for adaptive design

A central theme of this Research Topic is understanding the mechanisms and control principles that can guide the adaptive design of exoskeletons, as highlighted by several contributions spanning clinical feasibility, neurophysiological mechanisms, and integrative reviews. de Seta and Romeni provide a narrative review on closed-loop systems integrating exoskeletons with neuromodulation (e.g., spinal stimulation) and mobile brain imaging for individuals with neuromotor disorders, underscoring multisystem feedback and personalization as future standards. Hofstetter et al. describe a mixed-methods feasibility study of the HAL^®^ Lumbar Type exoskeleton in long-term care, designed to evaluate usability, acceptance, and its potential to reduce caregivers' lower back strain during physically demanding tasks. The study aims to provide evidence for integrating assistive exoskeletons into routine care settings to support both staff wellbeing and quality of patient care. Shamantseva et al. examine the effects of transcutaneous spinal cord stimulation on quiet standing in healthy participants—a mechanistic study emphasizing the importance of considering cognitive aspects in balance control and suggesting a potential synergy with exoskeleton training. Avaltroni, Cappellini et al. contribute a physiologically-grounded review of spinal motor maps, highlighting how neuromechanical principles can shape exoskeleton control paradigms and link motoneuron activity to gait mechanics, neuroprosthetics, and therapies for locomotor impairments, with applications in interpersonal coordination, gait assessment, and exoskeleton training.

## Pediatric cerebral palsy: adaptive biomechanics in natural terrain

Several contributions address pediatric cerebral palsy, highlighting how adaptive exoskeletons and biomechanical analyses can inform rehabilitation strategies across different levels of impairment and developmental stages. Tagoe et al. show that two sessions of untethered ankle exoskeleton training on varied terrain significantly improved assisted walking speed (+11 %) and unassisted speed (+8 %) in individuals with CP. Notably, stride length was the primary driver, yet muscle co-contraction remained unchanged—suggesting mechanical rather than neural adaptation during short training spans. The findings highlight the spatiotemporal benefits of an adaptive ankle exoskeleton for individuals with CP in real-world settings. Villani et al. analyze lower-limb EMG activity during exoskeleton-assisted walking in children, revealing distinctive developmental activation patterns that can inform age-adjusted control strategies. Even with full assistance, children's locomotor controllers interpret step-related afferent input to generate essential leg muscle activity, though with notable differences—reduced proximal muscle control, coactivation of lumbar and sacral motor pools, and weak distal extensor propulsion at push-off. This approach shows promise for both assessing exoskeleton performance and for guiding interventions that help children develop natural gait patterns and optimize systems for clinical use. Takahashi et al. report a case study of three children with cerebral palsy undergoing robot-assisted gait training with the pediatric-sized Hybrid Assistive Limb (HAL-2S). The intervention improved hip and knee extension during stance, enhanced muscle activation, and increased patient-reported performance and satisfaction, highlighting the feasibility and potential therapeutic value of HAL-assisted training in pediatric rehabilitation. In the other article, Avaltroni, Ivanenko et al. evaluate a reciprocating exoskeleton system in children with severe CP (GMFCS levels IV–V), reporting improved standing and walking capacity alongside specific kinematic adaptations. These findings underscore feasibility even in high-need pediatric cases, allowing walking and training at home in children with severe CP, enhancing development, social interaction, and endurance, while being well-received by families.

## Neurological injury: stroke and SCI rehabilitation

Advances in exoskeleton research for stroke and spinal cord injury demonstrate both clinical efficacy and broader functional benefits, as shown by recent trials and feasibility studies included in this Research Topic. Jin et al. report a randomized clinical trial combining unilateral lower-limb exoskeleton training with conventional therapy post-stroke. Participants achieved faster gait recovery and balance improvements vs. controls, highlighting clinical benefit. Nadorf et al. evaluate the ABLE personal-use exoskeleton in individuals with SCI for at-home and community activities, finding high feasibility and usability (in respect to independent donning, doffing, level of assistance, performance of basic and advanced exoskeleton skills, and participant and therapist satisfaction with the device), critical for real-world deployment. Hu et al. report on motor-complete SCI patients showing improvements in bowel function and cardiovascular measures with exoskeleton walking—suggesting systemic benefits beyond locomotor recovery.

## Healthy adults and military applications

Exoskeleton research in healthy adults also points to applications beyond rehabilitation, with implications for occupational and military settings. Nagaraja et al. present a wearable ankle exoskeleton (ExoBoot EB60) used by healthy adult men to carry 22.7 kg over 5 km. They report enhanced stride length, reduced trunk flexion, and hip kinetics -demonstrating benefits under load that could inform ergonomic and occupational use.

## Conclusion

This Research Topic advances our understanding of exoskeleton gait training across age groups and types of injuries. The Research Topic confirms functional gains and systemic benefits, elucidates biomechanically grounded control strategies, and pioneers deployment outside the lab. Yet, to transition from pilot to practice, larger, long-term, and context-rich studies are needed—underpinned by adaptable, personalized control systems. This body of work sets a strong foundation for collaborative progress among neuroscientists, clinicians, and engineers, toward rehabilitation interventions that are effective, empowering, and widely accessible.

